# Concurrent hypoglossal and phrenic nerve stimulation in patients with obstructive and treatment emergent central sleep apnea

**DOI:** 10.1007/s11325-023-02939-5

**Published:** 2023-11-06

**Authors:** Armin Steffen, Christoph Schöbel, Julia Vogler, Karl-Ludwig Bruchhage, Roland Richard Tilz

**Affiliations:** 1 Department of Otorhinolaryngology, University of Lübeck, University Hospital Schleswig-Holstein, Campus Lübeck, Ratzeburger Allee 160, 23538 Lübeck, Germany; 2 Faculty of Sleep Medicine and Telemedicine, West German Lung Center, University Medicine Essen - Ruhrlandklinik, University Duisburg-Essen, Duisburg, Germany; 3 Department of Rhythmology, University Heart Center Lübeck, University Hospital Schleswig-Holstein, Lübeck, Germany; 4 German Center for Cardiovascular Research (DZHK), Partner Site Hamburg/Kiel/Lübeck, Lübeck, Germany; 5 LANS Cardio, Hamburg, Germany

**Keywords:** Treatment emergent central sleep apnea, PAP failure, Sleep apnea, Phrenic nerve stimulation, Hypoglossal nerve stimulation

## Abstract

**Background:**

Patients with obstructive or central sleep apnea are primarily treated with positive airway pressure treatment. There are novel implantable options targeting either obstructive sleep apnea using hypoglossal nerve stimulation (HNS) or central sleep apnea using phrenic nerve stimulation (PNS).

**Methods:**

Patients with sleep apnea were implanted with both HNS and PNS devices, and their response to each therapy was monitored using home sleep tests as well as Epworth Sleepiness scale (ESS).

**Results:**

We evaluated our concurrent neurostimulation approach in two patients. Both patients were implanted with two neuromodulation devices: The first case suffered from treatment emergent central sleep apnea after HNS activation for primarily obstructive sleep apnea (apnea–hypopnea index/AHI 54/h). The central portion resolved under PNS (AHI 23.7/h). The second case suffered from predominantly central sleep apnea (AHI 82/h). Here, the PNS device was implanted first, resulting in a subsequent reduction of the central portion. The residual obstructive sleep apnea was addressed using HNS (AHI 5.4/h). No interaction between the HNS and PNS systems was noticed in either of the two patients.

**Conclusions:**

In selected cases, a concurrent treatment with hypoglossal and phrenic nerve stimulation may lead to improvement of sleep apnea and patient satisfaction in a safe manner.

## Introduction

Central and obstructive sleep apnea (CSA/OSA) are well treated in most cases using a variety of different positive airway pressure therapy (PAP) modalities. In case of intolerance or compliance issues, several second line treatment options are available for OSA — including hypoglossal nerve stimulation (HNS) in selected candidates. Recently, there has been an increase in the amount of available clinical evidence on HNS, including several long-term follow-up reports, randomized controlled trials, and registry analyses of more than 2000 patients [1, 2]. There have been reports of treatment emergent central sleep apnea (TeCSA) — alluding to initially diagnosed OSA which turns into a more central pattern under OSA therapies such as continuous PAP [3]. For HNS, TeCSA is described anecdotally [4]; however, no information is provided on the phenotype of patients who eventually develop TeCSA under OSA treatment. In case of TeCSA in patients treated with PAP, the alternatives are far less broad. The transvenous phrenic nerve stimulation (PNS) has been shown to be safe and also achieve a near elimination of the central component of the AHI in a randomized trial including 5-year follow-up assessments [5, 6].

For PAP patients, the development of TeCSA is a severe burden as the reasons for PAP intolerance are similar in CSA and OSA in clinical practice. Here, we present our approach in which concurrent neural stimulation was utilized to manage combined CSA and OSA as well as TeCSA.

## Patients and methods

Due to mandatory hospital COVID restrictions, sleep assessments were performed with home-sleep test such as polygraphy and peripheral arterial tonometry instead of polysomnography.

Data evaluation was done retrospectively.

## Results

### Patient 1: Hypoglossal first, followed by phrenic nerve stimulation

Patient 1 was a moderately overweight 52-year-old man (body mass index/BMI 28 kg/m^2^), diagnosed with PAP intolerance due to mask leaks (Table 1). After developing TeCSA under continuous PAP even after polysomnographic pressure tuning, adaptive servoventilation (ASV) was introduced. ASV resulted in similar mask problems as CPAP. No heart failure or abnormal heart rhythm was detected during the clinical assessment or after echocardiography. In 2016, the clinical evaluation before HNS implantation (Inspire Medical Inc.) showed an appropriate candidate after sleep endoscopy demonstrated no complete concentric soft palate collapse. The patient demonstrated an obstructive pattern of severe sleep apnea characterized by an apnea–hypopnea index (AHI) of 40/h (Table 1). The HNS therapy management included repeated polysomnographic titrations, home sleep tests, and sleep endoscopies with activated HNS and electrode configuration changes. The patient suffered from OSA in lower HNS voltages — or from TeCSA in higher HNS voltages, without any success when resorting to the intermediary voltage range. With increasing daytime sleepiness affecting his everyday life, a PNS (remedē, ZOLL Respicardia Inc) was implanted in 2020. After titration of both implanted systems and additional soft palate stiffening to further address persistent obstructions, sleep apnea severity decreased significantly (Table 1). The usage for HNS was 8.3 h per night, whereas for PNS, the therapy duration was 2.6 h per night.

**Table 1:** Patient’s sleep apnea characteristics before and after treatment

Case 1	Baseline	HNS only	HNS and PNS
AHI	40/h	54/h	24/h
cAHI	0/h	48/h	0.9/h
ODI	43/h	49/h	18/h
T90	Not given	58%	10%
Mean oxygen saturation	92%	88%	93%
Minimal oxygen saturation	62%	64%	70%
ESS	17	24	17
Case 2	Baseline	PNS only	PNS and HNS
AHI	82/h	50/h	5/h
cAHI	56/h	20/h	0/h
ODI	78/h	33/h	2/h
T90	Not given	0%	0%
Mean oxygen saturation	Not given	96%	96%
Minimal oxygen saturation	Not given	89%	93%
ESS	19	15	14

*AHI* apnea hypopnea index, *ODI* oxygen desaturation index, *T90* per centage of sleep below 90% oxygen saturation, *HNS* hypoglossal nerve stimulation, *PNS* phrenic nerve stimulation, *ESS* Epworth Sleepiness Scale

### Patient 2: PNS first, followed by HNS hypoglossal nerve stimulation

**Fig. 1:**
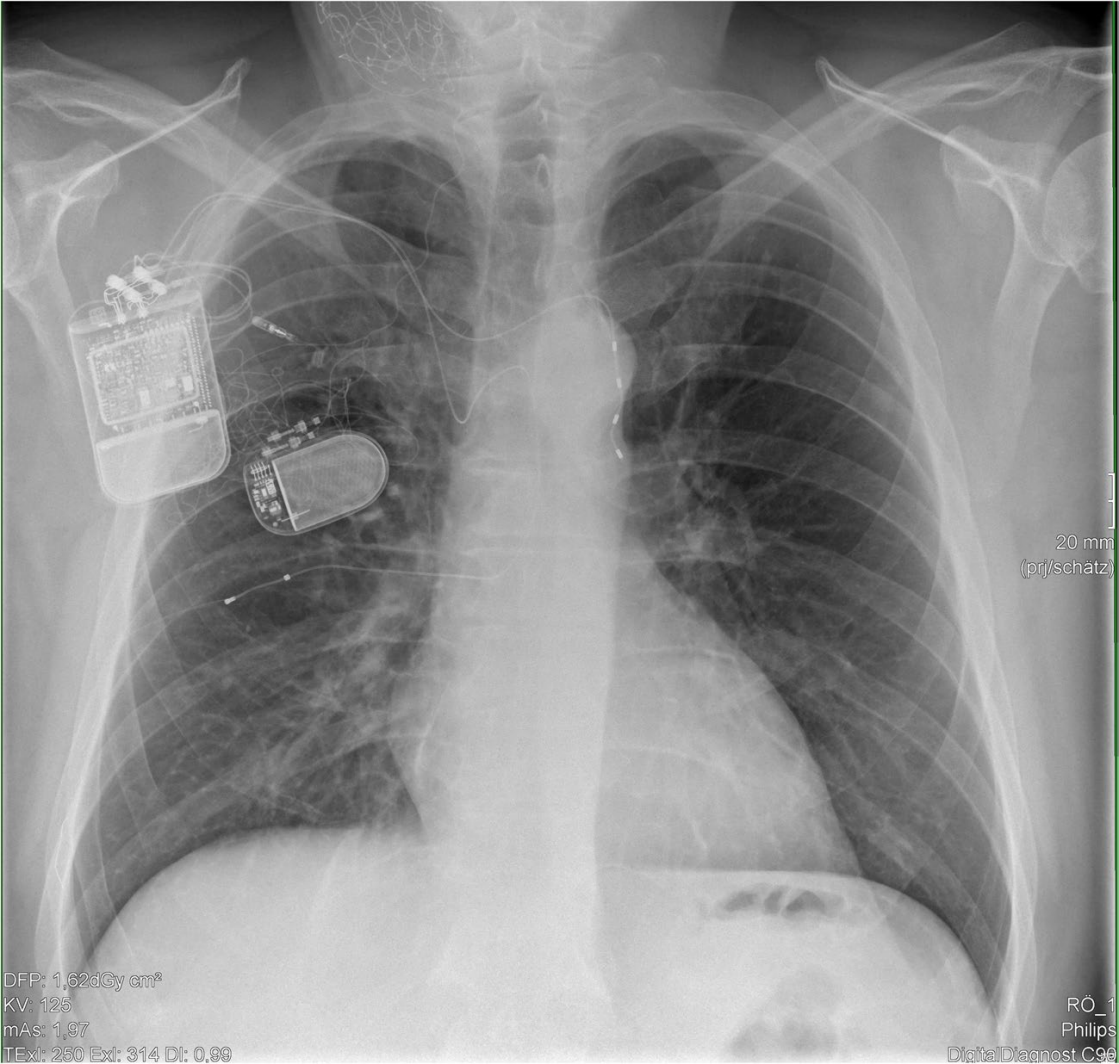
Combined hypoglossal and phrenic nerve stimulation devices in case 2 with central and obstructive sleep apnea

A 47-year-old man with class I obesity (BMI 31 kg/m^2^) could not tolerate the BiPAP therapy with unrestricted spontaneous breathing. The pressures were adjusted in light of severe sleep apnea with CSA, OSA, and mixed components. Several attempts to optimize therapy and increase the time under mask therapy and compliance were performed during polysomnography. The patient survived cerebellum malignancy in the late 1990s treated with surgery. Similar to the first case, no HF or abnormal heart rhythms were diagnosed. The main reason for PAP intolerance was the increased mask bandaging pressure used to address mask leaks, which lead to multiple patient complaints about massive headaches. In autumn 2020, PNS (remedē, Respicardia Inc) was implanted leading to a significant reduction of the CSA proportion (cAHI 56/h down to 19.6/h; Table 1). Subsequently, the patient demonstrated a visible improvement in daytime activity with subsequent weight loss. For the residual severe OSA, HNS was implanted in summer 2021 (Fig. 1). After therapy activation, the snoring was eliminated, and sleep disordered breathing indices were almost normalized (AHI 5/h; Table 1). The usage for HNS was 8.7 h per night, whereas for PNS, it was 2.6 h per night. His ESS scoring (Table 1) does not reflect his wife’s and physician’s perspective about the benefit in daily life with much increased activities and more social interactions.

## Discussion

This is the first report on concurrent phrenic and hypoglossal nerve stimulation in sleep apnea for patients who do not respond to PAP therapies, describing improved patient outcomes. In clinically complex sleep disorder cases, a combination of several approaches may be used — in both cases presented here, the central and obstructive sleep apnea components encountered by both patients were addressed using neurostimulation therapies. In both cases, daytime sleepiness improved but remained elevated (Table 1). In the HNS first-PNS second case (patient 1), the ESS daytime sleepiness improved with introduction of PNS therapy but remained equal to the baseline value. A potential explanation for this finding is that the baseline ESS was measured many years ago, and meanwhile, several unsuccessful and thereby discouraging attempts to optimize HNS were made.

For the second case, PNS first-HNS second (patient 2), the reported ESS of 14 points was higher than normal value. The patient still reported suffering from headaches after his brain tumor with interrupted sleep for many years because of apneas. This may explain the low PNS usage — the PNS system stops at night using actigraphy and positional sensing as the patients turns in bed or sits up when awake. Unfortunately, polysomnography was not consistently available due to COVID restrictions imposed during the pandemic and the effect of therapy was assessed using peripheral arterial tonometry throughout the follow-up.

Especially in cases with TeCSA, there is an important need for proper treatment beyond PAP modalities. The therapeutic outcomes presented in this report are relevant and may be considered when PAP therapies fail to address the underlying disease.

## Data Availability

Our manuscript has no associated data.
